# Simultaneous Wastewater‐to‐Hydrogen Upgrading via Mechanically Driven Piezocatalysis Over Ag‐ZnO Nanorods

**DOI:** 10.1002/advs.75354

**Published:** 2026-04-16

**Authors:** Yiqing Wei, Jianghai Huang, Yejunchi Zhang, Yang Wang, Dongmei Li, Chaogang Ban, Peng Yu, Jiangping Ma, Zhenxiang Cheng

**Affiliations:** ^1^ College of Physics and Optoelectronic Engineering Chongqing Normal University Chongqing China; ^2^ School of Integrated Circuits Chongqing University of Posts and Telecommunications Chongqing China; ^3^ Department of Applied Physics The Hong Kong Polytechnic University Hong Kong China; ^4^ Institute for Superconducting & Electronic Materials (ISEM) Australia Institute for Innovative Materials, Innovation Campus University of Wollongong North Wollongong New South Wales Australia

**Keywords:** dual redox utilization, mechanical energy conversion, piezocatalysis, wastewater‐to‐hydrogen upgrading, zinc oxide

## Abstract

The growing demand for sustainable hydrogen production has driven increasing interest in wastewater as a resource for simultaneous pollutant removal and energy recovery. However, most existing wastewater‐to‐hydrogen approaches rely on external electricity, light, or chemical agents, underutilizing mechanical energy and inherent charge complementarity. Here, a piezocatalytic upgrading strategy is developed to couple organic pollutant degradation with hydrogen evolution in a single mechanically driven system. Zinc oxide nanorods serve as the piezoelectric platform, while surface‐engineered silver regulates interfacial charge extraction and directional utilization. Under mechanical excitation, piezoelectric polarization generates complementary charges that drive oxidative pollutant degradation and reductive hydrogen evolution. Using rhodamine B as a model contaminant, the Ag‐modified ZnO nanorods deliver a 90.8% increase in hydrogen yield and a 339% enhancement in degradation kinetics compared to pristine ZnO. Notably, the system demonstrates versatile applicability across various classes of pollutants and real‐water matrices, maintaining efficient upgrading performance under ambient atmosphere and low‐intensity mechanical stirring. Combined experimental and theoretical results reveal that the Ag interface enhances charge separation, water activation, and hydrogen adsorption energetics. This work establishes a dual‐functional piezocatalysis paradigm for scalable wastewater‐to‐hydrogen upgrading.

## Graphical abstract

A piezocatalytic strategy enables simultaneous wastewater purification and hydrogen generation by harnessing mechanical energy. Complementary piezo‐induced charges are selectively directed toward oxidation and reduction pathways, achieving efficient pollutant degradation and continuous H_2_ evolution. This work establishes a scalable wastewater‐to‐hydrogen upgrading route, highlighting the potential of piezocatalysis for integrated energy and environmental applications.

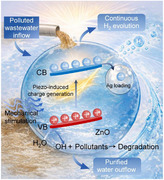

## 1 Introduction

Global efforts to address climate change and resource limitations are reshaping the conception of energy systems and environmental management [1, 2, 3]. Among emerging energy carriers, hydrogen has assumed a central role owing to its high energy density, clean utilization, and broad applicability [4, 5]. However, hydrogen production inherently depends on water, rendering water availability and sustainability an increasingly prominent constraint as green hydrogen technologies scale up. This intrinsic reliance underscores a close yet often underappreciated linkage between energy transition and water resource management.

In this broader context, the environmental remediation community has begun to reassess conventional wastewater treatment paradigms [6]. Historically, wastewater treatment has focused primarily on pollutant removal and regulatory compliance, with limited attention paid to the energetic potential contained in wastewater streams [7]. Such approaches generally require significant energy input while allowing the chemical energy stored in organic constituents to dissipate. As pressures on energy supply and water resources intensify, wastewater is gradually being reframed—from a passive waste stream into a potential participant in energy conversion processes [8]. Consequently, strategies that enable simultaneous pollution control and energy recovery are emerging as a timely and increasingly vital research direction in environmental catalysis.

To this end, various approaches have been explored to integrate wastewater treatment with hydrogen production, including biological routes [9], electrochemical systems [10, 11, 12], advanced oxidation processes [13, 14], and photo‐driven or photoelectrochemical technologies [15, 16]. By harnessing bioenergy, electricity, and solar energy, these strategies have demonstrated the feasibility of coupling wastewater remediation with hydrogen generation, collectively shaping the current landscape of wastewater energy valorization. From a broader energy perspective, however, a substantial fraction of low‐grade energy present in natural and engineered environments remains underexplored. Mechanical energy—ubiquitous in the form of fluid flow, waves, vibration, and shear—represents a largely untapped renewable resource [17, 18]. Compared with light and electricity, its application in driving catalytic chemical transformations, particularly for wastewater upgrading, remains at an early stage and warrants further investigation [19].

Piezocatalysis offers a distinctive mechanochemical pathway for directly converting mechanical energy into chemical reactivity [20, 21, 22]. Relying on the piezoelectric effect, piezocatalysts generate transient polarization charges under mechanical stimulation, enabling interfacial redox reactions without external light irradiation or electrical bias [23, 24]. Recent studies have demonstrated the broad applicability of piezocatalysis in environmental, biomedical, and energy‐related fields, with notable advances in organic pollutant degradation [25, 26], antimicrobial applications [27], carbon dioxide conversion [28, 29], and hydrogen evolution [30, 31, 32]. Despite these advances, efficient utilization of piezo‐induced charges remains a central challenge in practical piezocatalytic systems. Most reported studies tend to exploit piezo‐generated charges for a single functional objective [33], typically prioritizing either oxidative reactions or reductive processes, while the complementary charges are passively dissipated or intentionally suppressed [34]. Although such strategies simplify reaction control, they inevitably limit overall charge utilization efficiency and constrain the potential of piezocatalysis for integrated upgrading processes. The simultaneous generation of positive and negative charges under mechanical excitation creates an inherent opportunity for cooperative redox utilization. In wastewater systems, this redox complementarity naturally lends itself to coupled pollutant oxidation and fuel‐producing reduction. Thus, piezo‐induced charges can be selectively directed toward oxidation and reduction reactions within a single reaction environment, enabling concurrent pollutant degradation and fuel generation. Collectively, these attributes suggest that piezocatalysis holds promise as an integrated energy‐conversion platform for wastewater‐to‐hydrogen upgrading.

In this work, we demonstrate a piezocatalytic wastewater‐to‐hydrogen upgrading process in which organic pollutant removal and hydrogen generation are achieved simultaneously within a single reaction system. Zinc oxide nanorods serve as a model piezoelectric platform owing to their well‐defined structure and reliable piezoelectric response, while silver is introduced as an interfacial regulator to enhance charge extraction and directional utilization. In particular, Ag acts as an efficient electron sink due to its high conductivity and suitable work function, facilitating interfacial charge separation and promoting preferential electron transfer for hydrogen evolution, while enabling spatially differentiated utilization of complementary piezo‐induced charges. Using rhodamine B as a representative organic wastewater under mechanical excitation, we show that piezo‐induced positive and negative charges can be selectively steered toward oxidative degradation and reductive hydrogen evolution, respectively, thereby enabling a cooperative redox upgrading process. By integrating pollutant abatement and hydrogen production into a unified piezocatalytic framework, this work establishes a transferable paradigm for wastewater‐to‐hydrogen upgrading and highlights the broader potential of piezocatalysis as an integrated platform for energy and environmental conversion.

## 2 Results and Discussion

### 2.1 Construction and Characterization of Ag‐Engineered ZnO Piezocatalysts

**FIGURE 1:**
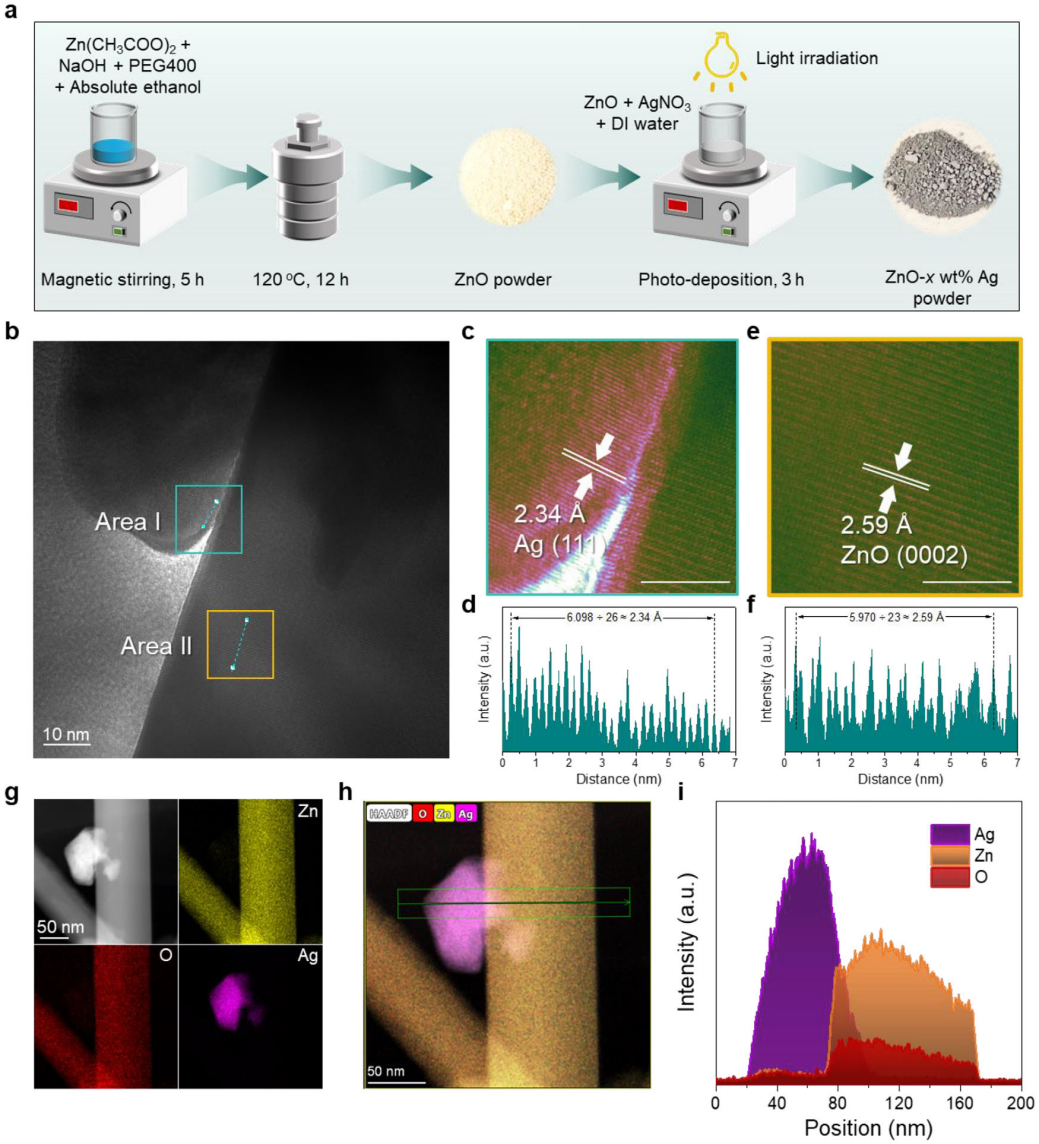
Synthesis and interfacial characterization of Ag‐engineered ZnO nanorods. (a) Schematic illustration of the synthesis process of Ag‐engineered ZnO nanorods. (b) HRTEM image of ZnO‐2wt.% Ag nanorods, with two representative regions (Areas I and II) highlighted for lattice analysis. Magnified HRTEM image of (c)Area I and (e) Area II. Scale bar: 5 nm. (d,f) Corresponding line profile analyses of two regions. (g) HAADF‐STEM image and elemental mapping of Zn, O, and Ag, demonstrating the spatial distribution of Ag species on ZnO nanorods. (h,i) Line‐scan elemental profile extracted from HAADF‐STEM mapping, showing the co‐localization of Ag at the nanorod surface.

The design of an efficient piezocatalyst for wastewater‐to‐hydrogen upgrading starts with the precise construction of interfacial sites capable of modulating the surface electronic environment. Toward this goal, silver species were introduced onto hydrothermally synthesized ZnO nanorods via a controlled photodeposition process (Figure 1a) [35]. This approach intentionally anchors Ag onto the nanorod surface, allowing electronic tuning while preserving the intrinsic wurtzite framework of ZnO. High‐resolution transmission electron microscopy (HRTEM) imaging confirms the successful decoration of Ag on the ZnO nanorods (Figure 1b). Two representative lattice regions (Areas I and II) were selected for detailed structural analysis. In Area I, the enlarged HRTEM image (Figure 1c) displays well‐resolved lattice fringes, and the corresponding line‐profile analysis (Figure 1d) gives an interplanar spacing of ∼2.34 Å, matching the (111) planes of metallic Ag and confirming the formation of surface‐deposited Ag nanodomains. In Area II (Figure 1e), the lattice spacing measured from the line profile fringing Figure 1f is ∼2.59 Å, characteristic of the (0002) planes of wurtzite ZnO. These observations demonstrate the coexistence of distinct Ag and ZnO crystalline domains, with Ag preferentially located on the surface rather than incorporating into the ZnO lattice. The ZnO backbone thus retains high crystallinity after Ag modification. Further evidence of the interfacial structure comes from high‐angle annular dark‐field scanning transmission electron microscopy (HAADF‐STEM) and elemental mapping (Figure 1g). Zn and O signals outline the ZnO nanorod framework, while Ag appears as discrete nanoscale domains distributed along the surface. Line‐scan elemental profiles (Figure 1h,i) reveal clear surface enrichment of Ag and its spatial correlation with the nanorod edges, consistent with preferential surface anchoring rather than bulk substitution. Together, these structural analyses confirm the successful construction of Ag‐engineered ZnO nanorods that maintain well‐preserved ZnO crystallinity while hosting surface‐localized Ag domains. This well‐defined architecture provides a robust material foundation for subsequent studies of electronic structure, interfacial properties, and piezocatalytic upgrading performance.

**FIGURE 2:**
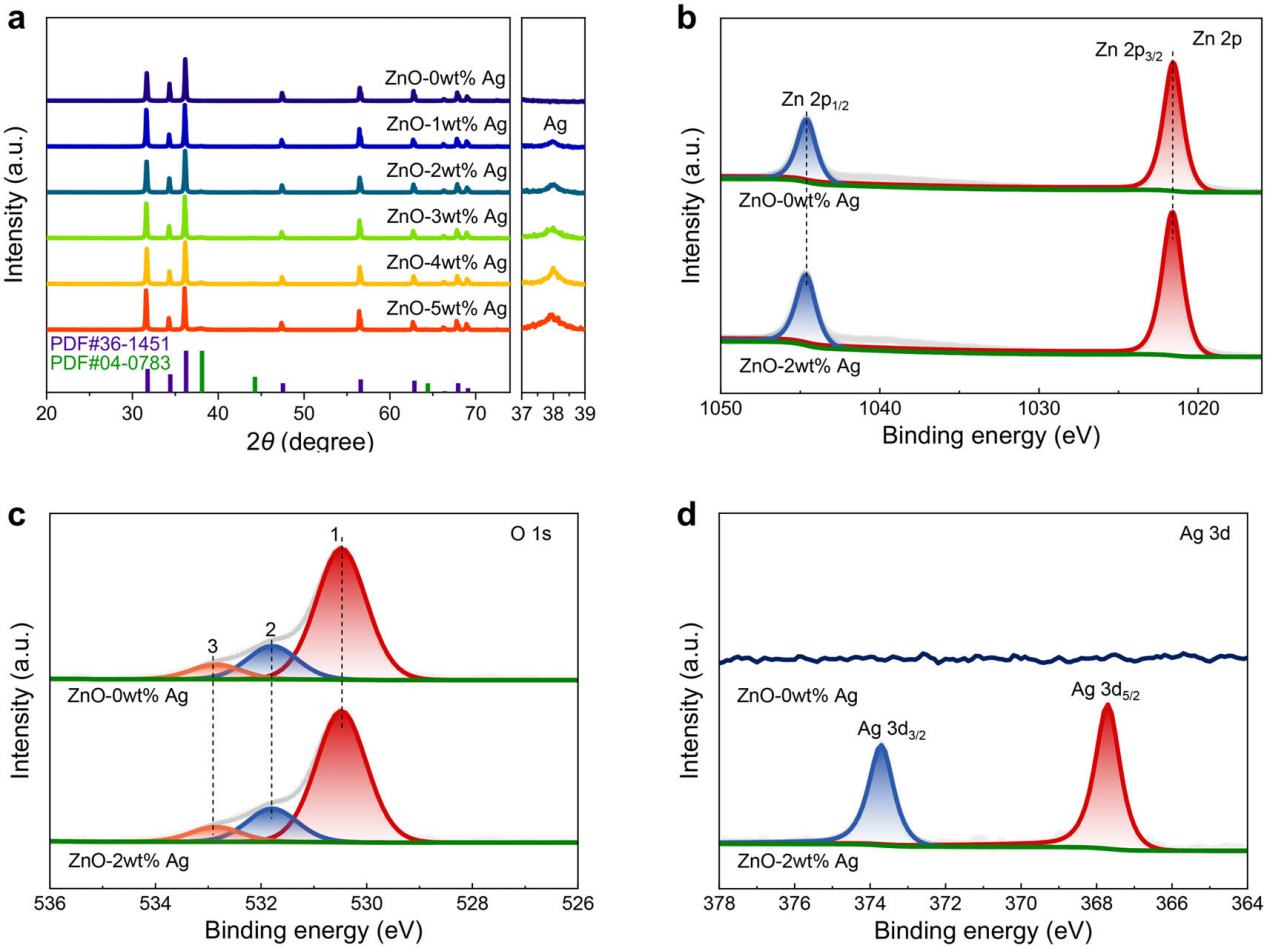
Structural and chemical characterization of Ag‐engineered ZnO nanorods. (a) XRD patterns of pristine ZnO and Ag‐modified ZnO samples with different Ag loadings. Right patten is the magnified diffraction (111) peak of Ag. High‐resolution XPS spectra of (b) Zn 2p, (c) O 1s, and (d) Ag 3d for ZnO‐0wt.% Ag and ZnO‐2wt.% Ag.

The crystalline structures of pristine and Ag‐modified ZnO nanorods were further examined by X‐ray diffraction (XRD) (Figure 2a). All samples exhibit characteristic diffraction peaks indexed to the hexagonal wurtzite ZnO phase (PDF#36‐1451) [36], confirming that the ZnO crystal structure remains intact after Ag modification. With increasing Ag loading, additional diffraction features corresponding to metallic Ag (PDF#04‐0783) gradually emerge and intensify [37, 38], verifying the successful incorporation of Ag species without inducing secondary oxide phases or distorting the ZnO host structure.

To further clarify the surface composition and chemical states, ZnO‐0wt.% Ag and ZnO‐2wt.% Ag was selected for X‐ray photoelectron spectroscopy (XPS) analysis (survey spectra in Figure S1). As shown in Figure 2b, the high‐resolution Zn 2p spectra exhibit two well‐defined peaks at 1021.6 and 1044.6 eV, corresponding to Zn 2p_3/2_ and Zn 2p_1/2_, respectively, characteristic of Zn^2+^ in the ZnO lattice [39]. Notably, no significant binding‐energy shift is observed upon Ag introduction, indicating that the local chemical environment and oxidation state of Zn remain unchanged. The O 1s spectra (Figure 2c) can be deconvoluted into three distinct components centered near 530.5, 531.8, and 532.8 eV, assigned to lattice oxygen, surface hydroxyl species, and adsorbed oxygen‐containing species (e.g., H_2_O or carbonates), respectively [40]. Similar to the Zn 2p spectra, the O 1s peak positions show negligible variation between ZnO‐0wt.% Ag and ZnO‐2wt.% Ag, suggesting that Ag modification does not alter the intrinsic Zn─O bonding or the oxidation state of the ZnO host. The successful deposition and chemical state of Ag are further corroborated by the high‐resolution Ag 3d spectra (Figure 2d). Two characteristic peaks located at 367.7 and 373.7 eV correspond to Ag 3d_5/2_ and Ag 3d_3/2_, respectively, indicative of metallic Ag^0^ species [41]. This confirms that Ag is deposited on the ZnO surface primarily as a metallic phase. The combined XRD and XPS results demonstrate that Ag is successfully introduced onto the ZnO nanorods as metallic Ag without disrupting the crystal structure or chemical states of the ZnO host. This leads to the formation of structurally well‐defined ZnO‐Ag nanocomposites with clear interfacial characteristics, providing a solid basis for the subsequent piezocatalytic studies.

### 2.2 Piezocatalytic Wastewater‐to‐Hydrogen Upgrading Conversion Enabled by Ag‐Engineered Interfaces

**FIGURE 3:**
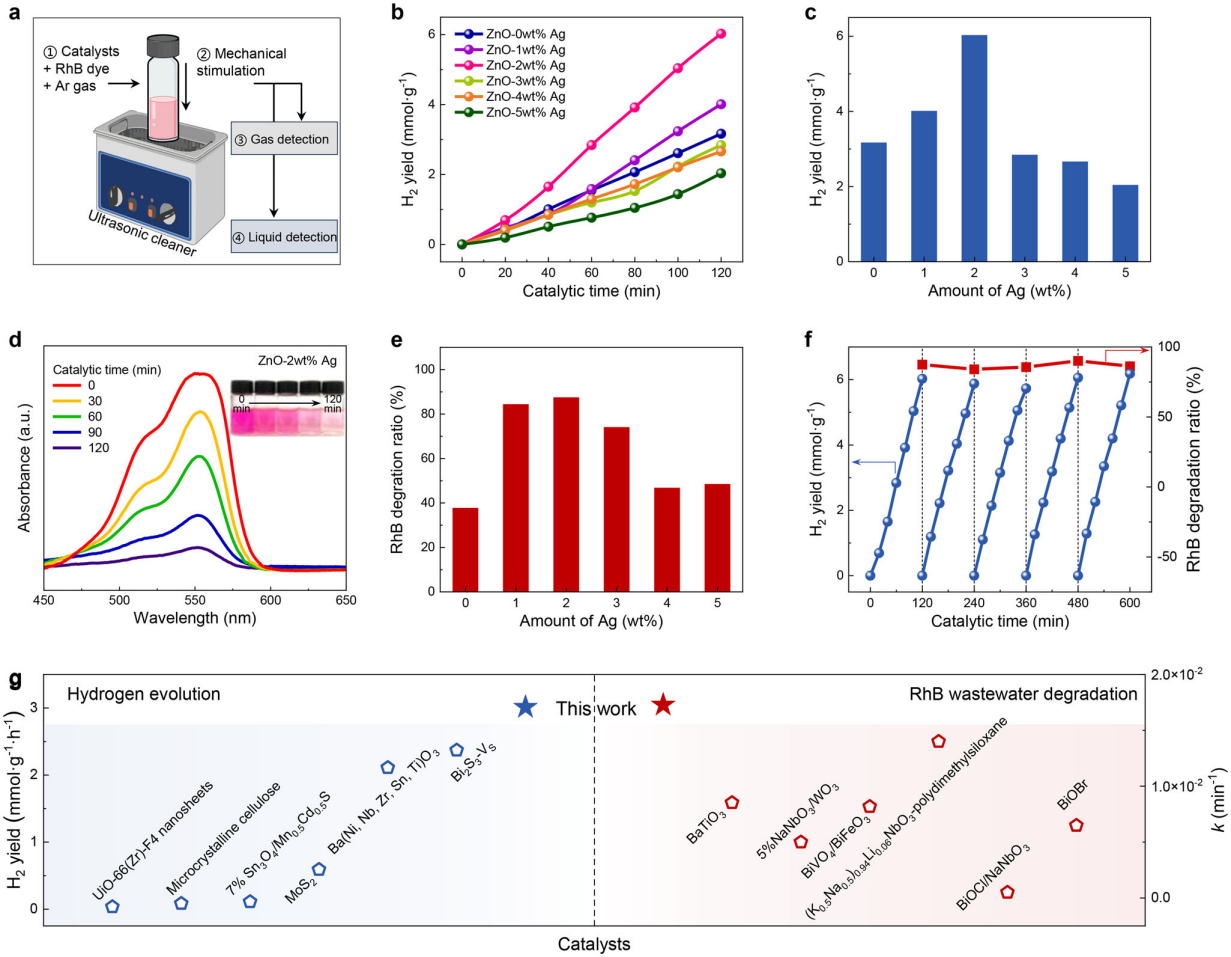
Piezocatalytic wastewater‐to‐hydrogen upgrading conversion performance of Ag‐engineered ZnO nanorods. (a) Schematic illustration of the ultrasonic‐driven piezocatalytic system enabling simultaneous wastewater degradation and hydrogen upgrading. (b) Time‐dependent hydrogen evolution over pristine ZnO and Ag‐modified ZnO nanorods. (c) Comparison of hydrogen production rates for different catalysts after 120 min. (d) UV–vis absorption spectra of RhB wastewater during piezocatalytic treatment using ZnO‐2wt.% Ag, with inset photographs showing progressive discoloration. (e) RhB wastewater degradation efficiencies after 120 min for ZnO and Ag‐engineered ZnO catalysts. (f) Cycling stability of simultaneous hydrogen evolution and RhB degradation using the ZnO‐2wt.% Ag catalyst. (g) Performance comparison of representative piezocatalytic systems reported in the literature for hydrogen evolution and organic pollutant degradation.

The piezocatalytic wastewater‐to‐hydrogen upgrading performance of the Ag‐engineered ZnO nanorods was evaluated using Rhodamine B dye (RhB, 10 mg L^−1^) as a model organic contaminant in an ultrasonic‐driven system (Figure 3a). Prior to ultrasonication, the catalyst was dispersed in the RhB solution and magnetically stirred for 1 h to establish adsorption–desorption equilibrium. Reactions were performed under an Ar atmosphere. Gaseous products were quantified by gas chromatography equipped with a self‐developed gas‐sampling system (Figure S2), while the degradation of RhB was monitored by UV–vis spectrophotometry. A recirculating water‐cooling setup was used to dissipate heat generated during ultrasonication, ensuring stable reaction conditions and minimizing thermal contributions.

Gas analysis confirms that H_2_ is the dominant product with only trace CO detected, corresponding a high H_2_ selectivity of over 97% (Figure S3). As shown in Figure 3b, the time‐resolved H_2_ evolution profiles for both pristine ZnO and ZnO‐*x*wt.% Ag catalysts increased steadily over 120 min of ultrasonication, indicating sustained catalytic activity under vibrational excitation. In contrast, control experiments performed without mechanical excitation show negligible RhB degradation and hydrogen generation (Figure S4), confirming that the catalytic process is governed by piezo‐induced charge generation. Hydrogen yields after 120 min are summarized in Figure 3c. Pristine ZnO produced delivers 3.16 mmol g^−1^, whereas Ag‐modified ZnO gave yields of 4.01, 6.03, 2.84, 2.66, and 2.04 mmol g^−1^ for Ag loadings of 1, 2, 3, 4, and 5wt.%, respectively. A clear volcano‐type dependence on Ag content was observed, with ZnO‐2wt.% Ag showing the highest hydrogen production rate— a 90.8% improvement over pristine ZnO. This volcano trend underscores the critical role of Ag loading in optimizing piezocatalytic performance. Moderate Ag deposition enhances the overall conductivity of the catalyst and facilitates charge migration and interfacial charge separation driven by piezoelectric polarization, thereby improving charge‐utilization efficiency [42, 43]. Conversely, excessive Ag loading can shield active surface sites, weaken effective interfacial polarization [44, 45], and introduce additional charge‐recombination centers, collectively suppressing piezocatalytic activity and leading to decreased hydrogen yield at higher loadings.

In parallel, the pollutant‐removal capability was evaluated by monitoring the degradation of RhB. Figure 3d shows the time‐dependent UV–vis absorption spectra of RhB treated with the representative ZnO‐2wt.% Ag catalyst. The characteristic absorption peak gradually diminished with prolonged ultrasonication, accompanied by a visible color change from deep pink to nearly colorless (inset photographs), confirming effective piezocatalytic degradation. UV–vis absorption spectra for other ZnO‐*x*wt.% Ag catalysts are provided in Figure S5. The degradation ratio *D* was calculated as *D* = (1 − *C*/*C*_0_) × 100% [46, 47], where *C*_0_ and *C* represent the absorbance at ∼554 nm at 0 and 120 min, respectively. As summarized in Figure 3e, Ag modification markedly improved degradation efficiency, with ZnO‐2wt.% Ag achieving the highest RhB removal of 87.4% after 120 min. Total organic carbon (TOC) analysis further reveals a mineralization efficiency of ∼70% under the same conditions (Figure S6), indicating that a substantial fraction of RhB is completely decomposed rather than merely decolorized. The simultaneous enhancement of hydrogen production and pollutant degradation underscores that Ag‐engineered interfaces effectively promote charge utilization along both reductive and oxidative pathways.

To further elucidate how the coupled redox performance is regulated, we investigated the effects of both mechanical excitation intensity and RhB concentration. As shown in Figure S7, increasing ultrasonic power (from 60 to 120 W) leads to a monotonic enhancement in hydrogen evolution, accompanied by a corresponding increase in RhB degradation efficiency (Figure S8). This trend indicates that stronger mechanical excitation promotes the generation of piezo‐induced charges, thereby simultaneously enhancing both reductive and oxidative reaction pathways. In contrast, increasing the initial RhB concentration leads to a decrease in hydrogen evolution (Figure S9), despite the presence of more oxidizable substrates. This behavior can be attributed to intensified competition for charge carriers at higher pollutant concentrations, where a larger fraction of piezo‐induced charges is consumed in oxidation processes rather than proton reduction. Taken together, these results reveal that the overall upgrading performance is governed by a cooperative interplay between charge generation and charge allocation: mechanical excitation controls the total amount of piezo‐induced charges, while substrate concentration dictates their distribution between oxidative degradation and reductive hydrogen evolution.

The operational stability of ZnO‐2wt.% Ag catalyst was evaluated through cycling hydrogen evolution tests (Figure 3f). The catalyst maintained a high level of hydrogen production level over five consecutive cycles, with only a slight decline upon repeated use. Post‐reaction characterizations confirmed structural robustness: XRD patterns before and after cycling showed no noticeable changes (Figure S10), indicating preservation of the ZnO crystal structure. HAADF‐STEM imaging and elemental mapping (Figure S11) further revealed that the nanorod morphology and surface‐distributed Ag domains remained intact after cycling, while line‐scan profiles (Figure S12) verified the stable spatial distribution of Ag along the ZnO nanorods. These results demonstrate good structural and interfacial stability of the Ag‐engineered ZnO nanorods under continuous ultrasonic excitation. Minor fluctuations in hydrogen evolution during cycling are attributed mainly to unavoidable catalyst loss during recovery or slight experimental variations, rather than intrinsic degradation, supporting the suitability of this system for sustained piezocatalytic upgrading.

To contextualize the performance of the present system, a comparative analysis with representative piezocatalytic systems from the literature was conducted. In addition to hydrogen evolution, pollutant degradation was quantified using the pseudo‐first‐order rate constant (*k*) [48, 49]. The kinetic plots of ln(*C*_0_/*C*) versus time for pristine ZnO and ZnO‐2wt.% Ag are provided in Figure S13. The rate constant for ZnO‐2wt.% Ag reached 0.01727 min^−1^, corresponding to a 339% enhancement relative to pristine ZnO, confirming significantly accelerated degradation kinetics enabled by Ag engineering. Figure 3g summarizes hydrogen yields and RhB degradation rate constants for various reported piezocatalytic systems (Tables S1 and S2). Overall, the Ag‐engineered ZnO nanorods developed here exhibit a competitive combination of hydrogen production activity and wastewater‐degradation kinetics, positioning this system within the performance range of known piezocatalysts and highlighting its balanced capability for concurrent hydrogen evolution and organic wastewater degradation under piezocatalytic conditions.

To evaluate the applicability of the proposed system beyond a model compound, we extended the piezocatalytic tests to representative organic pollutants with distinct chemical characteristics using ZnO‐2wt.% Ag catalyst, including a typical dye (methylene blue), an antibiotic (tetracycline), and an aromatic compound (phenol). In all cases (Figure S14), efficient degradation was achieved under identical conditions, accompanied by simultaneous hydrogen evolution, indicating that the coupled redox process is not limited to RhB. Furthermore, a mixed system containing multiple pollutants was constructed to better simulate complex wastewater conditions. The system maintained stable degradation performance and hydrogen production in this multi‐component environment, demonstrating its robustness against substrate diversity and competitive reactions. These results collectively confirm that the proposed strategy is broadly applicable across different classes of organic contaminants and remains operative under more realistic, compositionally complex conditions.

To further assess the feasibility of the system under realistic scenarios, we systematically investigated its performance under a range of practical conditions. The reaction proceeds efficiently under ambient atmosphere without inert gas purging (Figure S15), and can also be driven by low‐intensity mechanical inputs such as magnetic stirring (Figure S16), confirming that the process is not restricted to ultrasonic excitation. In addition, comparable activity was observed in real water matrices, including tap water and natural lake water (Figure S17), albeit with a slight decrease due to the presence of competing ions and impurities. The influence of key environmental parameters was also examined: increased salinity and solution conductivity facilitate charge migration and enhance catalytic performance (Figure S18), acidic conditions favor both pollutant degradation and hydrogen evolution (Figure S19), while elevated dissolved oxygen redistributes charge consumption between oxidation and reduction pathways (Figure S20). Notably, the overall activity decreases with increasing temperature (Figure S21), further confirming that the process is governed by mechanically induced charge generation rather than thermal effects. These results demonstrate that the proposed system is adaptable to diverse environmental conditions and maintains its functionality beyond idealized laboratory settings.

### 2.3 Interfacial Charge Dynamics and Mechanistic Origins of the Upgrading Conversion

**FIGURE 4:**
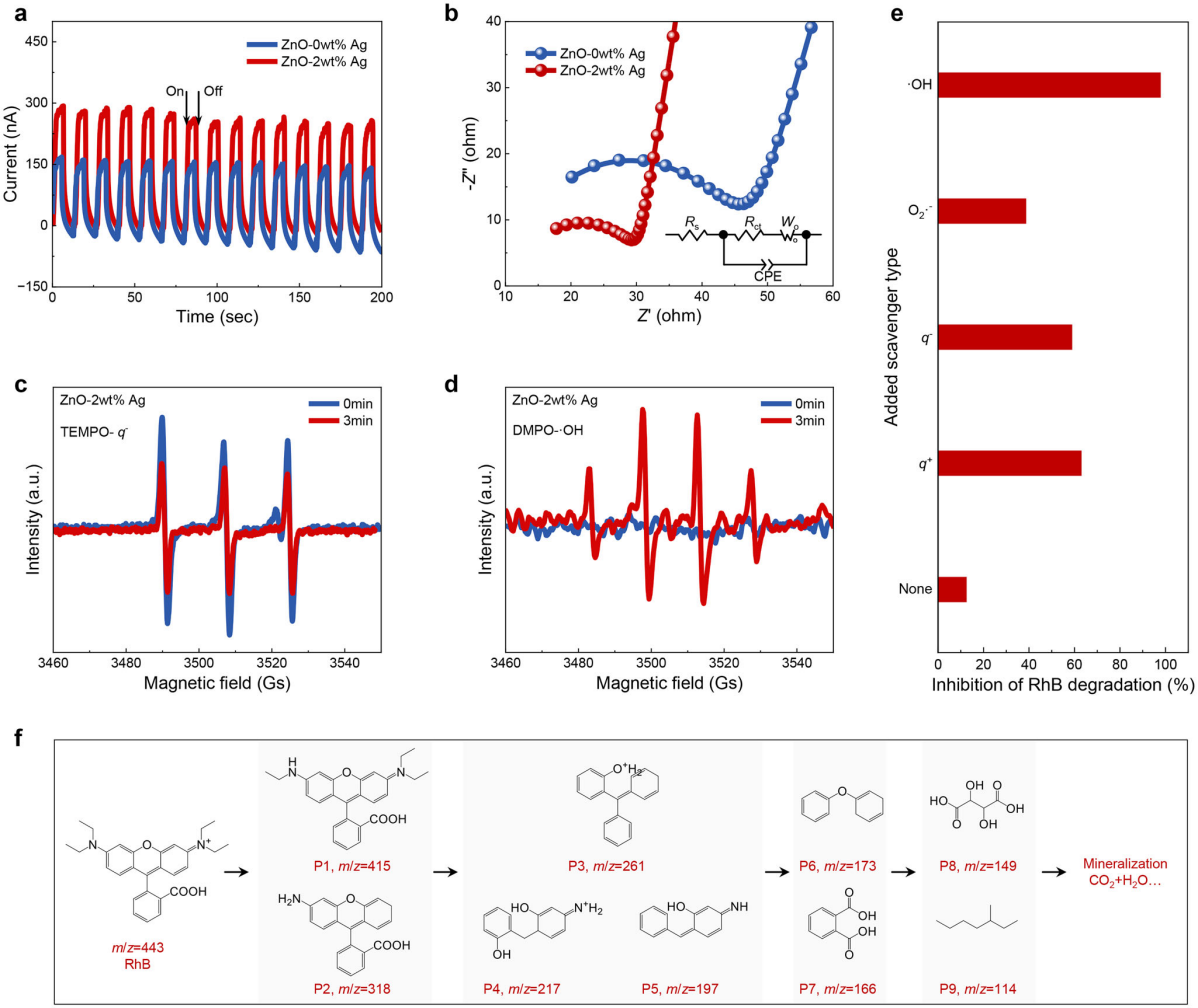
Mechanistic investigation of piezocatalytic wastewater‐to‐hydrogen upgrading conversion over Ag‐engineered ZnO nanorods. (a) Piezo‐current responses of pristine ZnO and ZnO‐2wt.% Ag under periodic mechanical excitation. (b) Electrochemical impedance spectroscopy (EIS) Nyquist plots, showing reduced charge‐transfer resistance for ZnO‐2wt.% Ag. EPR spectra of (c) *q* ^−^ and (d) ·OH captured using TEMPO and DMPO, respectively. (e) Trapping experiments indicating ·OH as the dominant reactive species involved in RhB wastewater degradation. (f) Plausible piezocatalytic degradation pathway of RhB wastewater over ZnO‐2wt.% Ag inferred from LC‐QTOF‐MS‐identified intermediates.

To elucidate the mechanistic origins of the enhanced wastewater‐to‐hydrogen upgrading enabled by Ag‐engineered interfaces, the interfacial charge dynamics and reactive species involved in the piezocatalytic process were systematically investigated. The piezoelectric charge generation and transport behavior of pristine ZnO and ZnO‐2wt.% Ag were first examined by piezo‐current response measurements under periodic mechanical excitation (Figure 4a). Compared with pristine ZnO, the ZnO‐2wt.% Ag showed a significantly stronger piezo‐current response, indicating more efficient generation and extraction of polarization‐induced charges [50]. This enhancement suggests that surface‐deposited Ag effectively facilitates charge migration across the ZnO interface, reducing charge accumulation and recombination under dynamic mechanical excitation. Electrochemical impedance spectroscopy (EIS) measurements further corroborated the improved interfacial charge‐transfer induced by Ag modification [51]. As shown in the Nyquist plots (Figure 4b), ZnO‐2wt.% Ag exhibited a lower charge‐transfer resistance than pristine ZnO (Table S3), confirming accelerated electron transport at the interface. Together, the piezo‐current and EIS results demonstrate that Ag‐engineered interfaces not only enhance polarization‐driven charge generation but also lower the kinetic barrier for interfacial charge‐transfer, thereby boosting overall charge‐utilization efficiency.

Based on these results, ZnO‐2wt.% Ag was selected for further electron paramagnetic resonance (EPR) analysis. The generation of piezo‐induced negative charges (*q* ^−^) was probed using 2,2,6,6‐tetramethylpiperidinooxy (TEMPO) as a spin probe [52]. Compared with the spectrum recorded before ultrasonication (Figure 4c), the TEMPO signal intensity decreased markedly after 3 min of ultrasonic excitation, indicating consumption of TEMPO radicals by piezo‐generated *q* ^−^ and confirming the dynamic formation of negative charges under mechanical stimulation. These accumulated negative charges furnish the reductive charge carriers required for hydrogen evolution. Subsequently, the formation of reactive oxygen species was examined using 5,5‐dimethyl‐1‐pyrroline N‐oxide (DMPO) as a spin‐trapping agent [53]. As shown in Figure 4d, no obvious EPR signal is observed without ultrasonication, whereas characteristic four‐line signals with a 1:2:2:1 intensity ratio emerged after 3 min of ultrasound. These signals can be unequivocally assigned to DMPO‐·OH adducts, confirming the generation of hydroxyl radicals during the piezocatalysis and providing the oxidative species responsible for RhB degradation.

To clarify the roles of different reactive species in the piezocatalytic degradation of RhB, scavenger experiments were performed (Figure 4e). Ethylenediaminetetraacetate disodium salt, potassium bromates, tert‐butanol, and p‐benzoquinone were used to trap positive charges (*q* ^+^), negative charges (*q* ^−^), hydroxyl radicals (·OH), and superoxide radicals (·O_2_^−^) [54, 55, 56], respectively; corresponding UV–vis absorption spectra are provided in Figure S22. The inhibition of RhB degradation was quantified as (1‐*D*) in the presence of each scavenger, with a higher value indicating a more critical role of the trapped species. As shown in Figure 4e, addition of the ·OH scavenger resulted in the most pronounced inhibition (∼97.9%), nearly suppressing RhB degradation. In contrast, other scavengers caused comparatively smaller inhibition effects, demonstrating that hydroxyl radicals play a predominant role in piezocatalytic RhB degradation, while other reactive species contribute to a lesser extent. The possible formation of ·O_2_^−^ under Ar‐saturated conditions is attributed to the reduction of trace dissolved O_2_ by piezo‐induced negative charges, where residual O_2_ may remain after purging or be generated in situ during oxidation processes [57].

To further trace the degradation evolution of RhB, reaction intermediates were identified by liquid chromatography‐quadrupole time‐of‐flight mass spectrometry (LC‐QTOF‐MS). After piezocatalytic treatment, the solution was centrifuged, and the supernatant was analyzed by LC‐QTOF‐MS. Based on the detected intermediates and their *m*/*z* values (Figures S23–S27), a plausible degradation pathway is proposed in Figure 4f. The degradation proceeds through several characteristic stages, consistent with previously reported RhB transformation in advanced oxidation systems [58, 59, 60]. Initially, RhB (*m*/*z* = 443) undergoes stepwise N‐deethylation, yielding dealkylated intermediates P1 (*m*/*z* = 415) and P2 (*m*/*z* = 318). With continued piezocatalytic treatment, these species undergo further oxidation and cleavage of the xanthene chromophore, producing smaller aromatic intermediates such as P3 (*m*/*z* = 261), P4 (*m*/*z* = 217), and P5 (*m*/*z* = 197). At later stages, ring‐opening reactions break down the aromatic framework, generating low‐molecular‐weight products P6 (*m*/*z* = 173), P7 (*m*/*z* = 166), P8 (*m*/*z* = 149), and P9 (*m*/*z* = 114). Ultimately, these fragments are gradually mineralized to CO_2_ and H_2_O under sustained oxidative conditions.

**FIGURE 5:**
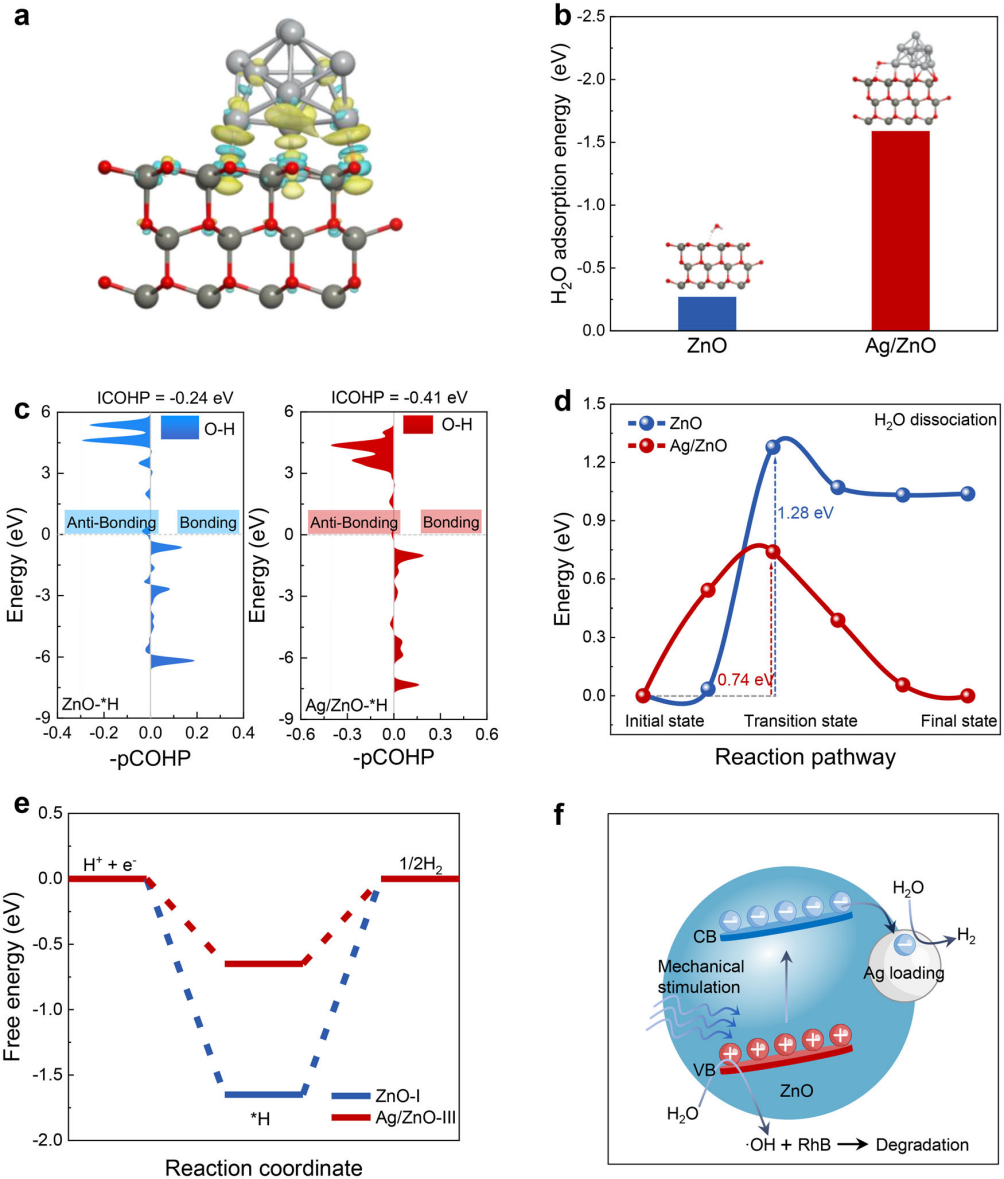
Theoretical insights into interfacial water activation and hydrogen evolution on Ag‐engineered ZnO nanorods. (a) Charge density difference of the Ag/ZnO interface, where yellow and cyan represent electron accumulation and depletion, respectively. Color code: Ag‐light gray, Zn‐gray, O‐red. (b) Comparison of H_2_O adsorption energies on pristine ZnO and Ag/ZnO surfaces, with representative optimized adsorption configurations shown as insets. (c) Projected crystal orbital Hamilton population (pCOHP) and corresponding integrated COHP (ICOHP) between adsorbates and surface atoms, with the Fermi level set to zero. (d) Calculated reaction energy profiles for water dissociation (H_2_O → ^*^H + ^*^OH) on pristine ZnO and Ag/ZnO surfaces. (e) Free‐energy diagrams of the hydrogen evolution reaction on ZnO and Ag/ZnO at different inequivalent adsorption sites. (f) Schematic illustration of mechanically driven piezocatalytic wastewater‐to‐hydrogen upgrading.

To gain mechanistic insight into how Ag‐engineered interfaces promote concurrent wastewater degradation and hydrogen evolution, density functional theory (DFT) calculations were performed to examine interfacial charge redistribution, water activation, and hydrogen evolution energetics on pristine ZnO and Ag‐modified ZnO surfaces (Figure 5). Figure 5a shows the charge‐density difference at the Ag/ZnO interface, revealing pronounced electron accumulation and depletion regions near the contact. This redistribution indicates enhanced electronic coupling between Ag and ZnO, providing a favorable pathway for polarization‐induced charge‐transfer under mechanical excitation. The strengthened interfacial electronic interaction aligns with the experimentally observed enhancement in piezo‐current response and reduced charge‐transfer resistance, suggesting that Ag acts as an effective mediator for extracting and transporting piezo‐induced charges, rather than serving as an independent catalytic phase.

Water adsorption, as the initial step governing both oxidative and reductive reaction pathways, was subsequently evaluated. The thermodynamically preferred adsorption configurations of H_2_O on pristine ZnO and Ag/ZnO were identified (Figure 5b; Figure S28). On pristine ZnO, H_2_O adsorbs primarily via hydrogen‐bonding interactions, with an adsorption energy of ‐0.27 eV. In contrast, the Ag/ZnO system exhibits pronounced chemisorption at interfacial sites, yielding a substantially higher adsorption energy of ‐1.59 eV at the most stable configuration. The markedly stronger water adsorption on Ag/ZnO indicates that interfacial sites effectively stabilize the H_2_O, facilitating subsequent activation. To gain deeper insights into the adsorption behaviors, the bonding characteristics were analyzed using projected crystal orbital Hamilton population (pCOHP) calculations (Figure 5c). The integrated COHP (ICOHP) value for the ZnO‐^*^H interaction is ‐0.24 eV, while a more negative value of ‐0.41 eV is obtained for the Ag/ZnO‐*H interaction, indicating strengthened interfacial bonding after Ag modification.

The energy barriers for ^*^H_2_O cleavage into ^*^H and ^*^OH (^*^H_2_O → ^*^H + ^*^OH) on ZnO and Ag/ZnO were also investigated (Figure 5d). Corresponding structural models of the initial, transition, and final states are provided in Figures S29 and S30. On pristine ZnO, water dissociation is endothermic with a barrier of 1.28 eV. In contrast, the presence of Ag not only makes the reaction exothermic but also lowers the energy barrier to 0.74 eV. The substantially reduced dissociation barrier on Ag/ZnO suggests that interfacial Ag sites facilitate O─H bond cleavage, promoting the formation of surface‐bound hydroxyl species (*OH). This energetically favorable formation of ^*^OH provides a mechanistic basis for the ·OH radicals detected by EPR, linking theoretical water activation to the oxidative degradation of organic pollutants.

To further understand the enhanced hydrogen evolution performance, the Gibbs free energy charge of adsorbed H (Δ*G*_H_) was systematically evaluated for both systems (Figure 5e). Structural optimization shows that the most stable hydrogen adsorption configuration on pristine ZnO localizes at a surface oxygen atom (ZnO‐I, Figure S31), exhibiting excessively strong ^*^H binding with a Δ*G*_H_ of ‐1.65 eV, which is thermodynamically unfavorable for efficient H_2_ desorption. On Ag/ZnO, five inequivalent adsorption sites were identified (Figure S32). Among them, the Ag/ZnO‐III site shows the most balanced hydrogen adsorption, with Δ*G*_H_ = ‐0.65 eV—substantially closer to the thermoneutral condition required for efficient hydrogen evolution. Free‐energy diagrams for all considered sites are summarized in Figure S33, confirming that Ag modification introduces energetically favorable sites for H_2_ evolution.

Taken together, these theoretical results establish a coherent mechanistic picture: Ag‐engineered interfaces simultaneously enhance interfacial charge‐transfer, promote water adsorption and dissociation to generate surface ^*^OH species, and optimize hydrogen adsorption energetics. The dual promotion of oxidative (^*^OH‐mediated degradation) and reductive (H_2_ generation) pathways provides a unified energetic explanation for the experimentally observed piezocatalytic wastewater‐to‐hydrogen upgrading behavior enabled by Ag‐modified ZnO nanorods. This integrated charge–reaction coupling is schematically summarized in Figure 5f.

## 3 Conclusion

In summary, this work demonstrates a versatile piezocatalytic approach that achieves synchronous organic pollutant degradation and hydrogen evolution directly driven by mechanical energy. Employing ZnO nanorods as the piezoelectric host, silver surface engineering serves as an interfacial regulator to enhance charge extraction and steer the utilization of piezo‐induced complementary charges toward coupled oxidation and reduction pathways. The optimized ZnO‑2wt.% Ag catalyst exhibits a hydrogen yield of 6.03 mmol g^−1^—a 90.8% improvement over pristine ZnO—along with 87.4% removal of RhB within 120 min. Crucially, this dual‐functional framework demonstrates robust applicability across diverse pollutant classes (including antibiotics and aromatics) and natural water matrices, maintaining efficient upgrading performance under ambient atmosphere and low‐intensity mechanical stirring. Mechanistic investigations combined with DFT calculations reveal that Ag‑engineered interfaces facilitate interfacial charge‐transfer and separation, promote water activation to furnish ·OH radicals for oxidation, and optimize hydrogen adsorption energetics. This study establishes a transferable framework for dual‐functional piezocatalysis, reframing wastewater as a redox‐participating feedstock for sustainable energy recovery. Looking forward, this framework holds promise for application in flow‐through wastewater systems and integration with existing treatment trains, enabling mechanically powered wastewater‐to‐hydrogen energy recovery

## Conflicts of Interest

The authors declare no conflicts of interest.

## Supporting information




**Supporting File**: advs75354‐sup‐0001‐SuppMat.docx

## Data Availability

The data that support the findings of this study are available from the corresponding author upon reasonable request.
