# Supplementary material for: Simultaneous Wastewater‐to‐Hydrogen Upgrading via Mechanically Driven Piezocatalysis Over Ag‐ZnO Nanorods
Source: Adv Sci (Weinh). 2026 Apr 16:e75354. Online ahead of print. doi: 10.1002/advs.75354 (PMC13335725; doi:10.1002/advs.75354)
Supplement: Supplementary file 1 — Supporting File: advs75354‐sup‐0001‐SuppMat.docx [file ADVS-9999-e75354-s001.docx]

**Supplementary information for**

**Simultaneous Wastewater-to-Hydrogen Upgrading via Mechanically Driven Piezocatalysis over Ag-ZnO Nanorods**

Yiqing Wei^a,†^, Jianghai Huang^a,†^, Yejunchi Zhang^a^, Yang Wang^b^, Dongmei Li^a^, Chaogang Ban^c^, Peng Yu^a,*^, Jiangping Ma^c,*^, Zhenxiang Cheng^d,*^

^a^ College of Physics and Optoelectronic Engineering, Chongqing Normal University, Chongqing 401331, China

^b^ School of Integrated Circuits, Chongqing University of Posts and Telecommunications, Chongqing 400065, China

^c^ Department of Applied Physics, The Hong Kong Polytechnic University, Hong Kong, 999077, China

^d^ Institute for Superconducting & Electronic Materials (ISEM), Australia Institute for Innovative Materials, Innovation Campus, University of Wollongong, Squires Way, North Wollongong, NSW 2500, Australia

^†^ These authors contributed equally to this work.

* Correspondence and request for materials should be addressed to P. Yu (Email: pengyu@cqnu.edu.cn), J. Ma (Email: jiangping.ma@polyu.edu.hk) and Z. Cheng (Email: cheng@uow.edu.au).

**Table of content**

[Experimental Section 3](#_Toc216364744)

[Materials 3](#_Toc216364745)

[Synthesis of ZnO Nanorods 3](#_Toc216364746)

[Synthesis of ZnO-*x*wt% Ag Catalysts 3](#_Toc216364747)

[Materials Characterization 4](#_Toc216364748)

[Piezocatalytic Hydrogen Evolution Tests 4](#_Toc216364749)

[Piezocatalytic Dye Degradation Tests 5](#_Toc216364750)

[Electron Paramagnetic Resonance (EPR) Measurements 5](#_Toc216364751)

[LC-QTOF Analysis of RhB Degradation Intermediates 6](#_Toc216364752)

[Piezochemical Measurements 6](#_Toc216364753)

[Calculation Details 7](#_Toc216364754)

[Supplementary Figures 8](#_Toc216364755)

[Supplementary Tables 41](#_Toc216364756)

[References 44](#_Toc216364757)

# Experimental Section

# Materials

Analytical-grade reagents were used throughout this work. Zinc acetate (Zn(CH_3_COO)_2_·2H_2_O), sodium hydroxide (NaOH), polyethylene glycol 400 (PEG-400), silver nitrate (AgNO_3_), Rhodamine B (RhB), and anhydrous ethanol (C_2_H_5_OH) were purchased from Aladdin Biochemical Technology Co., Ltd. and used without further purification. These chemicals were employed as precursors and auxiliary reagents in the hydrothermal synthesis of ZnO nanorods and the subsequent Ag photodeposition process.

# Synthesis of ZnO Nanorods

ZnO nanorods were synthesized via a hydrothermal method [1]. In a typical procedure, 1.1 g of zinc acetate Zn(CH_3_COO)_2_·2H_2_O and 4.0 g of NaOH were dissolved in a mixed solution containing 30 mL of absolute ethanol and 7.5 mL of PEG-400. The precursor mixture was magnetically stirred for 5 h until fully homogenized, and then transferred into a Teflon-lined stainless-steel autoclave. The sealed autoclave was heated at 120 °C for 12 h to facilitate the growth of ZnO nanorods. After naturally cooling to room temperature, the resulting product was collected by centrifugation at 8000 rpm for 3 min and washed several times with deionized water and ethanol to obtain purified ZnO nanorods.

# Synthesis of ZnO-*x*wt% Ag Catalysts

Ag-loaded ZnO catalysts with different silver weight percentages (*x*wt%) were prepared via a photodeposition method. Typically, 100 mg of the as-synthesized ZnO nanorods were dispersed in a mixture containing *x* mL of AgNO_3_​ aqueous solution (Ag: 1 mg mL^-1^) and (50-*x*) mL of deionized water, yielding a total volume of 50 mL. The suspension was transferred into a 100 mL quartz reaction vessel equipped with a magnetic stir bar, purged with O_2_​, and then sealed. The reaction mixture was irradiated with a 300 W Xe lamp for 3 h under continuous stirring to drive the photoreduction of Ag^+^ onto the ZnO surface. After completion of photodeposition, the resulting ZnO-*x*wt% Ag catalysts were allowed to settle, and the supernatant was removed. The solids were collected by centrifugation using a single 10 mL centrifuge tube per sample, followed by repeated washing with deionized water and ethanol. The purified products were finally dried to obtain ZnO-*x*wt% Ag catalysts.

# Materials Characterization

The crystal structures of the ZnO-*x*wt% Ag samples were analyzed by X-ray diffraction (XRD) using a PANalytical X’Pert diffractometer equipped with Cu Kα radiation (λ = 1.5406 Å) operated at 40 kV and 40 mA. The morphology and microstructure were examined by transmission electron microscopy (TEM), high-resolution TEM (HRTEM), high-angle annular dark-field scanning TEM (HAADF-STEM), and energy-dispersive X-ray spectroscopy (EDS) using a Thermo Fisher Scientific Talos F200S G2 microscope. Surface chemical composition and valence states were characterized by X-ray photoelectron spectroscopy (XPS, Thermo Fisher Scientific ESCALAB 250Xi), with all binding energies calibrated to the C 1s peak at 284.80 eV.

# Piezocatalytic Hydrogen Evolution Tests

For piezocatalytic hydrogen evolution measurements, 10 mg of catalyst was dispersed in 20 mL of pollutant solution (10 mg L^-1^, unless otherwise specified), including RhB, methylene blue, tetracycline, phenol, or their mixed solution, and transferred into a 100 mL borosilicate reaction tube. The suspension was magnetically stirred for 1 h to establish adsorption-desorption equilibrium prior to ultrasonic excitation. Subsequently, Ar was purged through the reactor to remove dissolved and headspace gases, after which the tube was sealed. The sealed reactor was then placed at a fixed position in a Jiemeng ultrasonic bath (80 kHz, 120 W) to initiate the piezocatalytic reaction. Gas samples were collected every 20 min over a total reaction duration of 2 h. At each interval, 0.5 mL of headspace gas was withdrawn using a self-developed automated gas sampling device and analyzed using a gas chromatograph (GC7900, Techcomp) to quantify the piezo-generated hydrogen. Other possible gaseous products were further analyzed using a Shimadzu Nexis GC-2030 gas chromatograph. A circulating water-cooling system was employed throughout the ultrasonic process to minimize temperature fluctuations and suppress thermal effects.

# Piezocatalytic Dye Degradation Tests

After establishing adsorption-desorption equilibrium, a 1 mL aliquot of the pollutant solution was withdrawn and centrifuged to remove suspended catalysts. The resulting supernatant was analyzed using a UV-vis spectrophotometer (Shanghai Spectrum Instruments Co., Ltd.) to determine the initial concentration. Following the piezocatalytic reaction for 120 min, another 1 mL aliquot was collected, centrifuged, and subjected to UV-vis analysis to obtain the final concentration.

For the ZnO-2wt% Ag catalyst, time-dependent degradation was additionally examined. A 1 mL aliquot was taken every 30 min, centrifuged to remove the solid catalysts, and analyzed by UV-vis spectroscopy to monitor the concentration change of RhB during the piezocatalytic process. The degree of mineralization was evaluated by measuring the total organic carbon (TOC) during the reaction using a TOC analyzer (vario TOC select, Elementar Analysensysteme GmbH, Germany).

To further identify the reactive species involved during piezocatalytic degradation, scavenger experiments were conducted under the same reaction conditions using ZnO-2wt% Ag. RhB solutions were supplemented with different scavengers at a concentration of 1 mmol L^-1^, including ethylenediaminetetraacetate disodium salt (*q*^+^), potassium bromates (*q*^−^), tert-butanol (·OH), and p-benzoquinone (·O_2_^−^). For each scavenger, 1 mL aliquots were collected before ultrasonication and after 120 min of reaction, centrifuged, and analyzed by UV-vis spectroscopy to evaluate the dye concentration and identify the dominant reactive species.

# Electron Paramagnetic Resonance (EPR) Measurements

EPR measurements were carried out using a Chinainstru & Quantumtech (Hefei) EPR200-Plus spectrometer operating in continuous-wave X-band mode. All experiments were performed using the ZnO-2wt% Ag catalyst under identical ultrasonic conditions with ultrapure water as the reaction medium. The only variable between measurements was the choice of spin-trapping agent. To detect piezo-induced negative charges (*q*^−^), 5 mg of ZnO-2wt% Ag was dispersed in 1 mL of 0.1 mM TEMPO aqueous solution. The interaction between TEMPO and piezo-generated *q*^−^ leads to the formation of TEMPO^−^, which can be detected by EPR. For hydroxyl radical (·OH) detection, 5 mg of ZnO-2wt% Ag was mixed with 1 mL of water containing 30 μL of DMPO to form the DMPO-·OH adduct. In both measurements, the suspensions were subjected to ultrasonic vibration (Jiemeng ultrasonic cleaner, 80 kHz, 120 W) for 0 min and 3 min, and EPR spectra were recorded immediately after each time point by transferring aliquots into capillary tubes inserted into quartz EPR tubes.

# LC-QTOF-MS Analysis of RhB Degradation Intermediates

The degradation intermediates of RhB during the piezocatalytic process were analyzed using liquid chromatography-quadrupole time-of-flight mass spectrometry (LC-QTOF-MS, Agilent QTOF 6545, Agilent Technologies). After piezocatalytic treatment, reaction solutions were collected, centrifuged to remove suspended catalyst particles, and the supernatant was subjected to LC-QTOF analysis. The high-resolution mass spectra obtained from the QTOF detector were used to identify the molecular fragments and propose the possible degradation pathway of RhB.

# Piezochemical Measurements

Piezo-current measurements and electrochemical impedance spectroscopy (EIS) were carried out using a CHI-760E electrochemical workstation (Chenhua, China). All electrochemical tests were performed in a standard three-electrode configuration, where a catalyst-coated glassy carbon electrode, a carbon rod, and a Hg/HgO electrode served as the working, counter, and reference electrodes, respectively.

To prepare the working electrode, catalyst ink was first formulated by dispersing 5 mg of catalyst in a mixture of 400 μL ethanol, 100 μL deionized water, and 20 μL Nafion solution, followed by ultrasonic treatment for 30 min. Subsequently, 10 μL of the ink was drop-cast onto the glassy carbon electrode and dried at room temperature. All measurements were conducted in 0.4 M Na_2_SO_4_ electrolyte.

# Calculation Details

All spin-polarized density functional theory (DFT) calculations in this study were performed by using the Vienna Ab initio Simulation Package [2, 3]. The electron-ion interaction was described by the projector-augmented wave method [4]. The electron exchange-correlation energy was treated with the Perdew-Burke-Ernzerhof functional within the generalized gradient approximation [5]. A plane-wave cutoff energy of 500 eV was applied to ensure computational accuracy and proper convergence [6]. Dipole correction was adopted in this study to improve the description of the asymmetrical configuration [3]. Transmission electron microscopy analysis indicates that the ZnO(0001) facet is the predominantly exposed surface. Moreover, previous studies confirm that the [0001] crystallographic direction of ZnO aligns with its spontaneous polarization [7], with the (0001) facet being the most stable surface [8, 9]. Accordingly, a 3-layer ZnO(0001) slab with a (3 × 3) supercell was constructed. The vacuum thickness of 15 Å was used to avoid interactions between periodic images. Γ-centered k-point grids of 3 × 3 × 1 and 5 × 5 × 1 were employed for geometry optimization and electronic structure calculations, respectively. The convergence criteria for energy and residual force were set to 10^−5^ eV and 0.02 eV/Å, respectively.

The adsorption energies of molecules (*E*_ads_) can be calculated as:

*E*_ads_ = *E*_mol+slab_ – *E*_slab_ – *E*_mol_

where *E*_mol+slab_ and *E*_slab_ represent the total energies of the slab with and without the adsorbed molecule, respectively. *E*_mol_ is the total energy of the isolated molecule.

The Gibbs free energy (*G*) can be calculated as:

*G* = *E* + *ZPE* ‒ *T*S

where *E* is the total energy of systems, *ZPE* is the zero-point energy, *S* is the entropy, and *T* is the temperature (298.15 K). The change in free energy (Δ*G*) is correspondingly given by Δ*G* = Δ*E* + Δ*ZPE* – *T*Δ*S*. The zero-point energies and entropies for adsorbed systems were obtained from vibrational frequency calculations. The entropies of free molecules were taken from the NIST database [10].

# Supplementary Figures

**Figure S1.** XPS survey spectra of pristine ZnO (ZnO-0wt% Ag) and Ag-modified ZnO (ZnO-2wt% Ag).


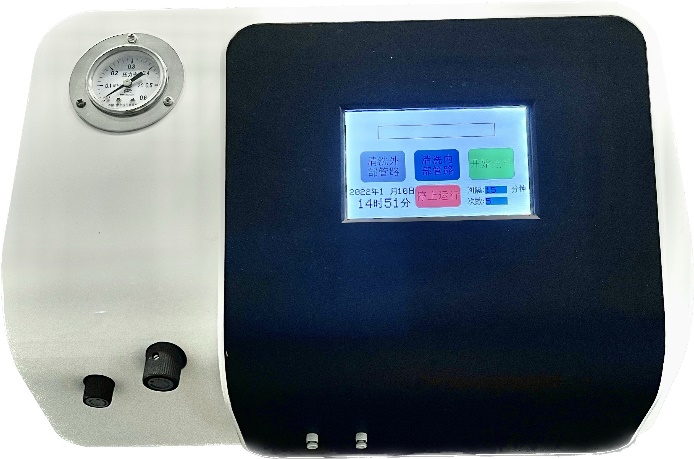


**Figure S2.** The self-developed automatic online gas sampling system.

**Figure S3.** Analysis of gaseous products during the piezocatalytic reaction.

**Figure S4.** Control experiments without ultrasonication. (**a**) UV–vis absorption spectra of RhB solution in the presence of ZnO-2wt% Ag without ultrasonication. (**b**) Time-dependent H_2_ evolution under identical conditions without ultrasonication.

**Figure S5.** UV–vis absorption spectra of RhB wastewater before (0 min) and after 120 min piezocatalytic reaction using ZnO-*x*wt% Ag catalysts (*x* = 0, 1, 3, 4, 5).

**Figure S6.** Total organic carbon (TOC) during the piezocatalytic degradation of RhB.

**Figure S7.** Ultrasonic power dependent hydrogen evolution performance of ZnO-2wt% Ag. (a) Time-resolved H_2_ evolution over ZnO-2wt% Ag catalysts under different ultrasonic powers. (b) Comparison of hydrogen production yields after 120 min of piezocatalytic reaction at different ultrasonic powers.

**Figure S8.** Ultrasonic power dependent piezocatalytic degradation of RhB over ZnO-2wt% Ag. UV–vis absorption spectra of RhB solution before (0 min) and after 120 min piezocatalytic treatment at ultrasonic powers of (a) 60 W and (b) 90 W, respectively. (c) RhB degradation efficiencies after 120 min as a function of ultrasonic power.

**Figure S9.** Effect of RhB concentration on piezocatalytic hydrogen evolution.

**Figure S10.** XRD patterns of ZnO-2wt% Ag catalysts before and after piezocatalytic hydrogen evolution cycle tests.


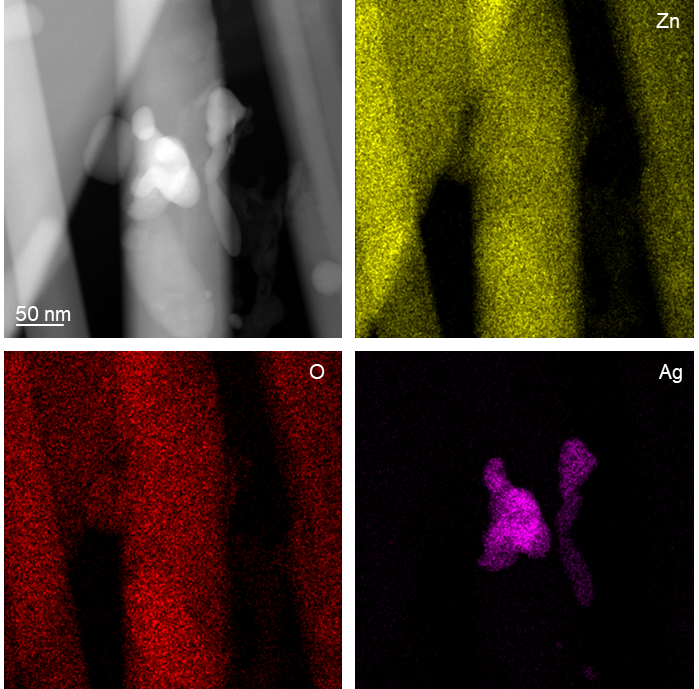


**Figure S11.** HAADF-STEM image and elemental mapping of ZnO-2wt% Ag catalysts after piezocatalytic hydrogen evolution cycle tests.


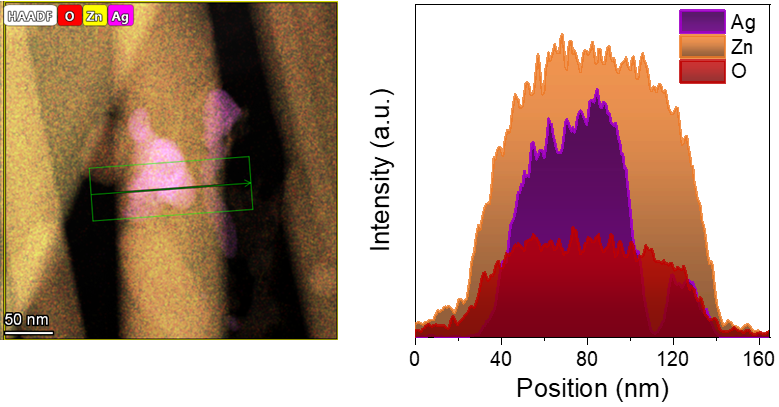


**Figure S12.** Line-scan elemental profile extracted from HAADF-STEM mapping of ZnO-2wt% Ag catalysts after piezocatalytic hydrogen evolution cycle tests.

**Figure S13.** Piezocatalytic degradation kinetics of RhB over Ag-engineered and pristine ZnO nanorods under ultrasonic excitation. (**a**, **c**) Time-dependent normalized concentration change (*C*/*C*_0_) of RhB during piezocatalytic degradation over ZnO-2wt% Ag and pristine ZnO, respectively. (**b**, **d**) Corresponding pseudo-first-order kinetic analyses of RhB degradation over ZnO-2wt% Ag and pristine ZnO, respectively, based on linear fittings of ln(*C*_0_/*C*) versus reaction time.

**Figure S14.** Versatile pollutant degradation and coupled hydrogen evolution over Ag–ZnO piezocatalyst. UV–vis absorption spectra of (**a**) methylene blue (MB), (**b**) tetracycline, (**c**) phenol and (**d**) mixed solution containing RhB, MB, tetracycline, and phenol before and after 120 min reaction. Insets in (**a**) and (**d**) show the corresponding color change. (**e**) Corresponding H_2_ evolution performance during degradation of different pollutants and their mixture.

**Figure S15.** Pollutant degradation and hydrogen evolution under ambient atmosphere without inert gas purging. (**a**) UV–vis absorption spectra of RhB wastewater at different reaction times under air atmosphere (without Ar purging). (**b**) Corresponding time-dependent normalized concentration change (*C*/*C*_0_) of RhB during piezocatalytic degradation. (**c**) Simultaneous H_2_ evolution performance under the same conditions.

**Figure S16.** Pollutant degradation and hydrogen evolution driven by mechanical stirring. (**a**) UV–vis absorption spectra of RhB wastewater at different reaction times under magnetic stirring. (**b**) Corresponding time-dependent normalized concentration change (*C*/*C*_0_) of RhB during piezocatalytic degradation. (**c**) Simultaneous H_2_ evolution performance under the same conditions.

**Figure S17.** Pollutant degradation and hydrogen evolution in real water matrices. (**a**) UV–vis absorption spectra of RhB dissolved in tap water before and after 120 min reaction. (**b**) Corresponding H_2_ evolution performance in tap water. (**c**) UV–vis absorption spectra of RhB dissolved in natural lake water (San Chun Lake, Chongqing Normal University) before and after reaction. Inset shows the sampling location. (**d**) Corresponding H_2_ evolution performance in lake water.

**Figure S18.** Effect of NaCl concentration on pollutant degradation and hydrogen evolution. (**a**–**c**) UV–vis absorption spectra of RhB in the presence of NaCl with concentrations of 0.1, 0.3, and 0.5 mol·L^-1^, respectively, at different reaction times. (**d**) Corresponding time-dependent normalized concentration change (*C*/*C*_0_) of RhB under different NaCl concentrations. (**e**) H_2_ evolution performance as a function of catalytic time under varying NaCl concentrations.

**Figure S19.** Effect of pH on pollutant degradation and hydrogen evolution. (**a**, **b**) UV–vis absorption spectra of RhB at different reaction times under acidic (pH = 3) and alkaline (pH = 11) conditions, respectively. (**c**) Corresponding time-dependent normalized concentration change (*C*/*C*_0_) of RhB under different pH conditions (pH = 3, 7, and 11). (**d**) H_2_ evolution performance as a function of catalytic time at different pH values.

**Figure S20.** Effect of dissolved oxygen concentration on pollutant degradation and hydrogen evolution. (**a**, **b**) UV–vis absorption spectra of RhB at different reaction times under elevated dissolved O_2_ concentrations, respectively. (**c**) Corresponding time-dependent normalized concentration change (*C*/*C*_0_) of RhB under different dissolved O_2_ concentrations. (d) H_2_ evolution performance as a function of catalytic time under varying dissolved O_2_ levels.

**Figure S21.** Effect of temperature on pollutant degradation and hydrogen evolution. (**a**, **b**) UV–vis absorption spectra of RhB at different reaction times under 40 °C and 60 °C, respectively. (**c**) Corresponding time-dependent normalized concentration change (*C*/*C*_0_) of RhB at different temperatures. (**d**) H_2_ evolution performance as a function of catalytic time at different temperatures.

**Figure S22.** UV-vis absorption spectra of RhB solutions at 0 min and 120 min during piezocatalytic degradation over ZnO-2wt% Ag catalysts in the presence of different scavengers.

**Figure S23.** LC–QTOF–MS analysis of initial RhB wastewater prior to piezocatalytic treatment.

**Figure S24.** LC–QTOF–MS spectra of RhB degradation intermediates at stage I during piezocatalytic treatment.

**Figure S25.** LC–QTOF–MS spectra of RhB degradation intermediates at stage II during piezocatalytic treatment.

**Figure S26.** LC–QTOF–MS spectra of RhB degradation intermediates at stage III during piezocatalytic treatment.

**Figure S27**. LC–QTOF–MS spectra of RhB degradation intermediates at stage IV during piezocatalytic treatment.


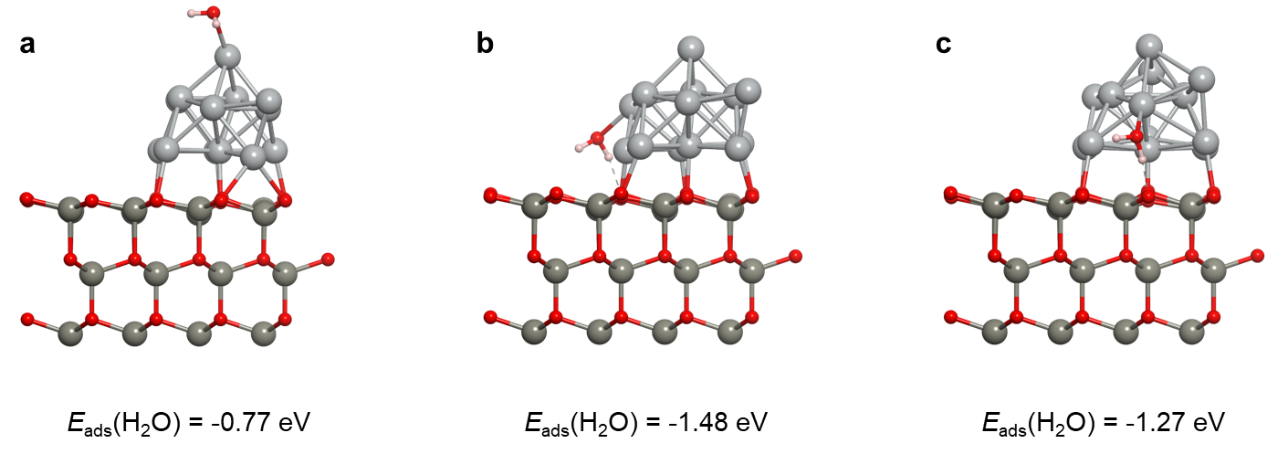


**Figure S28.** H_2_O adsorption configurations on Ag/ZnO at possible inequivalent sites.


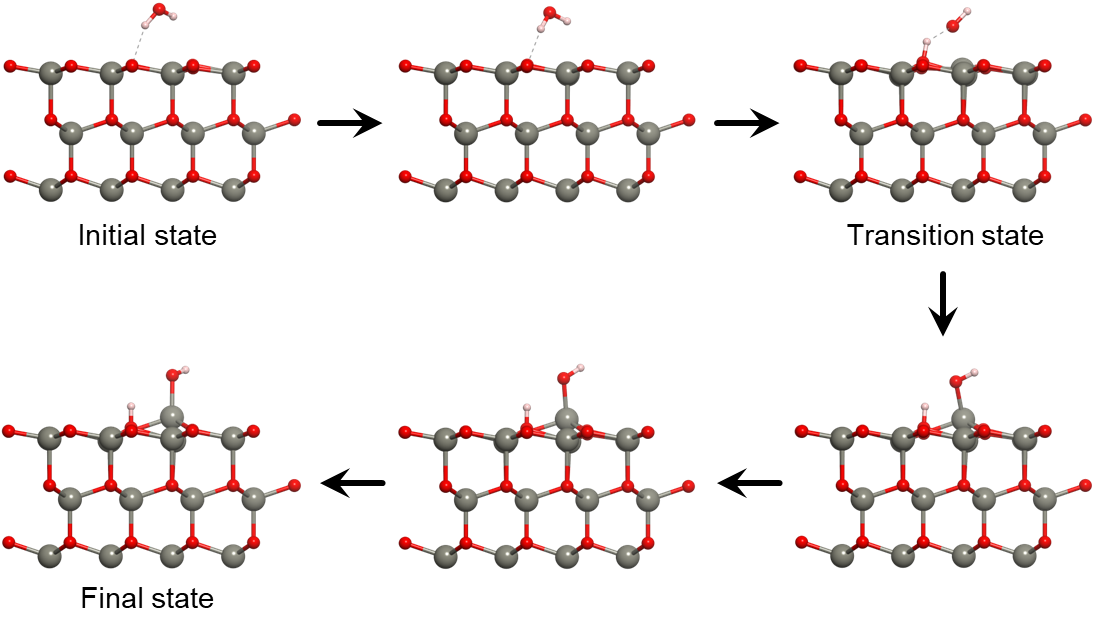


**Figure S29**. Structures of each state for water dissociation on ZnO.


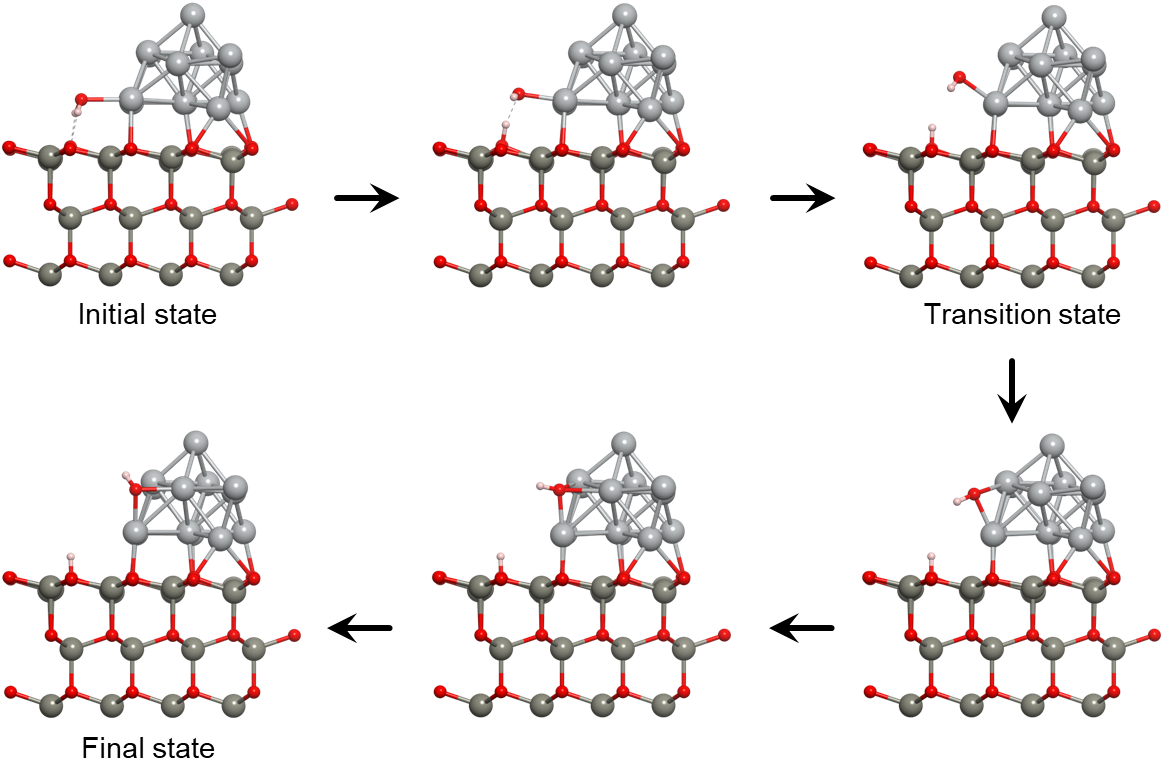


**Figure S30.** Structures of each state for water dissociation on Ag/ZnO.


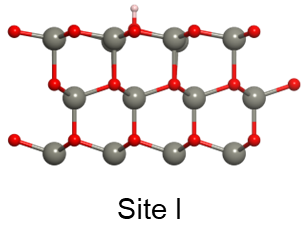


**Figure S31.** H adsorption configurations on ZnO.


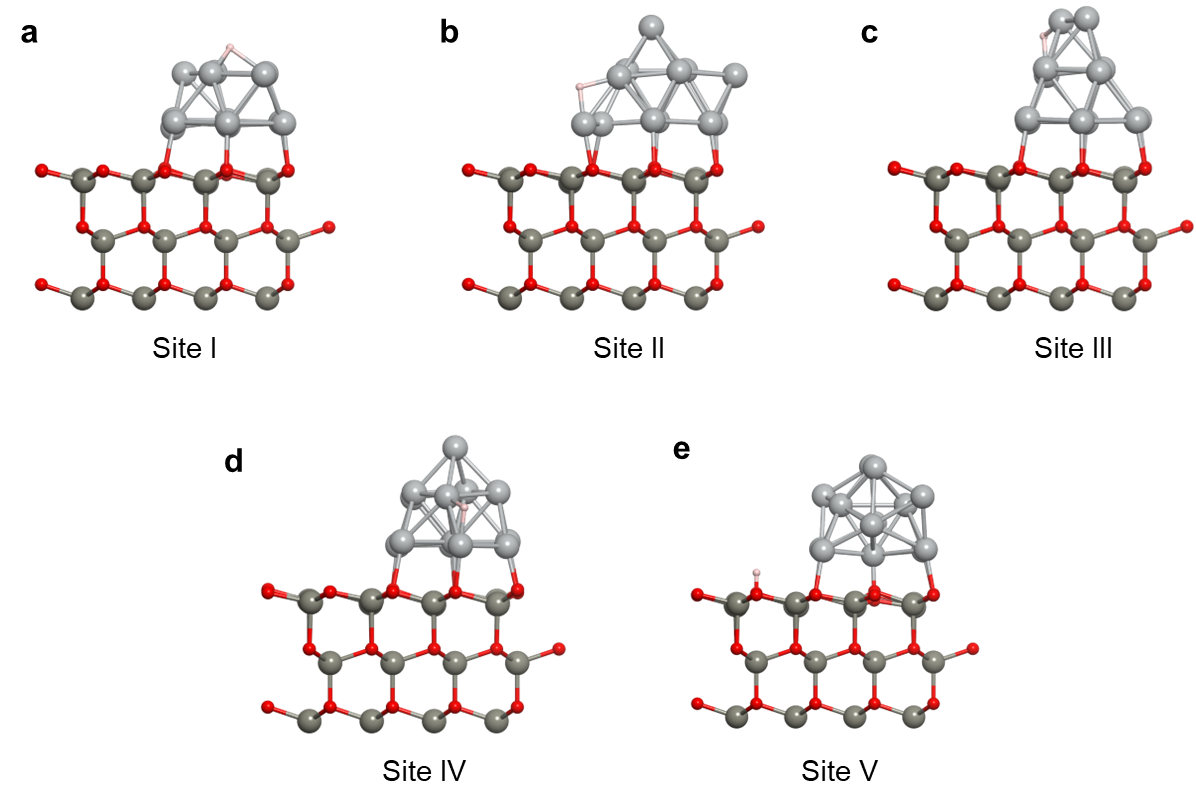


**Figure S32.** H adsorption configurations on Ag/ZnO at possible inequivalent sites.

**Figure S33.** Free energy diagram of hydrogen evolution reaction on Ag/ZnO at possible inequivalent sites.

# Supplementary Tables

**Table S1.** Comparison of catalytic performances for hydrogen evolution of catalysts in recently reported works and this study.

| Catalyst | Catalytic type | Solution | H_2_ yield (μmol·g^-1^·h^-1^) | Year | Ref. |
| --- | --- | --- | --- | --- | --- |
| ZnO-2wt% Ag | Piezo | RhB, 10 mg/L | 3012.94 |  | This work |
| ZnO/porous nickel foam | Piezo-photo | 5 mM Na_2_SO_3_ and 5 mM Na_2_S solution | 1020 | 2026 | [11] |
| ZnO/SrTiO_3_ | Piezo | 5% triethanolamine (TEOA) solution at pH 9 | 1265 | 2025 | [12] |
| MoS_2_/ZnO/CuFe_2_O_4_ | Piezo-photo | 10 vol % methanol–water mixture solution | 3963 | 2025 | [13] |
| ZnO/MoS_2_ | Piezo-photo | Nitenpyram solution | 746.56 | 2024 | [14] |
| Bi_2_S_3_-V_S_ | Piezo | 1.0 M Na_2_S and Na_2_SO_3_ aqueous solution | 2370 | 2025 | [15] |
| Ba(Ni, Nb, Zr, Sn, Ti)O_3_ | Piezo | 25 vol% methanol aqueous solution | 2110 | 2025 | [16] |
| MoS_2_ | Piezo | Seawater | 590.36 | 2025 | [17] |
| 7% Sn_3_O_4_/Mn_0.5_Cd_0.5_S | Piezo | 0.35 M Na_2_S + 0.25 M Na_2_SO_3_ aqueous solution | 109.95 | 2024 | [18] |
| Microcrystalline cellulose | Piezo | 10 vol% methanol aqueous solution | 84.47 | 2023 | [19] |
| UiO-66(Zr)-F4 nanosheets | Piezo | 0.15 M Na_2_SO_3_ aqueous solution | 35.7 | 2022 | [20] |

**Table S2.** Comparison of catalytic performances for wastewater degradation of catalysts in recently reported works and this study.

| Catalyst | Dosage | Catalytic type | Pollutant (mg/L) | *k* (min^-1^) | Degradation efficiency | Year | Ref. |
| --- | --- | --- | --- | --- | --- | --- | --- |
| ZnO-2wt% Ag | 0.5 g/L | Piezo | RhB, 10 mg/L | 0.01727 | 87.41%, 120 min |  | This work |
| ZnO@PVDF | - | Piezo-photo | MO, 5 mg/L | 0.0160 | 98.09%, 360 min | 2026 | [21] |
| ZnO-GO | ~1.43 g/L | Piezo | RhB, 10 mg/L | 0.0047 | 91%, 180 min | 2025 | [22] |
| g-C_3_N_4_/Ag/ZnO | 0.5 g/L | Piezo-photo | RhB, 10 mg/L | 0.0210 | 99%, 120 min | 2024 | [23] |
| Bi_0.5_Na_0.5_TiO_3_/ZnO | 1 g/L | Piezo-photo | RhB, 5 mg/L | 0.0180 | - | 2023 | [24] |
| g-C_3_N_4_/Ag/ZnO | 1 g/L | Piezo-photo | RhB, 10 mg/L | 0.0190 | 89%, 180 min | 2022 | [25] |
| BaTiO_3_ | 0.2 g/L | Piezo | RhB, 5 mg/L | 0.00852 | 90.2%, 90 min | 2024 | [26] |
| 5%NaNbO_3/_WO_3_ | 0.25 g/L | Piezo-photo | RhB, 10 mg/L | 0.00500 | 73.7%, 120 min | 2023 | [27] |
| BiVO_4_/BiFeO_3_ | 2g/L | Piezo | RhB, 5 mg/L | 0.00818 | ≈60%, 120 min | 2023 | [28] |
| (K_0.5_Na_0.5_)_0.94_Li_0.06_NbO_3_-polydimethylsiloxane | - | Piezo | RhB, 5 mg/L | ≈ 0.014 | - | 2023 | [29] |
| BiOCl/NaNbO_3_ | 1 g/L | Piezo | RhB, 5 mg/L | 0.00050 | 9.9%, 100 min | 2022 | [30] |
| BiOBr | 1 g/L | Piezo | RhB, 10 mg/L | 0.00649 | - | 2019 | [31] |

**Table S3.** Resistance data calculated from EIS.

| Catalyst | *R*_s_ (Ω) | *R*_ct_ (Ω) |
| --- | --- | --- |
| ZnO-0wt% Ag | 14.75 | 21.27 |
| ZnO-2wt% Ag | 12.23 | 18.61 |

# References

1. J. Ma, J. Ren, Y. Jia, Z. Wu, L. Chen, N.O. Haugen, H. Huang, Y. Liu, “High Efficiency Bi-Harvesting Light/Vibration Energy Using Piezoelectric Zinc Oxide Nanorods for Dye Decomposition”. *Nano Energy* 62 (2019): 376-383.

2. G. Kresse, J. Furthmüller, “Efficient Iterative Schemes for AB Initio Total-Energy Calculations Using A Plane-Wave Basis Set”. *Physical Review B* 54 (1996): 11169-11186.

3. G. Kresse, J. Furthmüller, “Efficiency of AB-Initio Total Energy Calculations for Metals and Semiconductors Using a Plane-Wave Basis Set”. *Computational Materials Science* 6 (1996): 15-50.

4. P.E. Blöchl, “Projector Augmented-Wave Method”. *Physical Review B* 50 (1994): 17953-17979.

5. J.P. Perdew, K. Burke, M. Ernzerhof, “Generalized Gradient Approximation Made Simple”. *Physical Review Letters* 77 (1996): 3865.

6. Y. Wang, J. Meng, S. Jing, K. Wang, C. Ban, Y. Feng, Y. Duan, J. Ma, L. Gan, X. Zhou, “Origin of Bismuth‐Rich Strategy in Bismuth Oxyhalide Photocatalysts”. *Energy & Environmental Materials* 6 (2023): e12432.

7. D. Segets, L. Martinez Tomalino, J. Gradl, W. Peukert, “Real-Time Monitoring of the Nucleation and Growth of ZnO Nanoparticles Using an Optical Hyper-Rayleigh Scattering Method”. *Journal of Physical Chemistry C* 113 (2009): 11995-12001.

8. X. Xiong, Y. Wang, J. Ma, Y. He, J. Huang, Y. Feng, C. Ban, L.-Y. Gan, X. Zhou, “Oxygen Vacancy Engineering of Zinc Oxide for Boosting Piezo-Electrocatalytic Hydrogen Evolution”. *Applied Surface Science* 616 (2023): 156556.

9. M. Ge, H. Wu, L. Niu, J. Liu, S. Chen, P. Shen, Y. Zeng, Y. Wang, G. Zhang, J. Jiang, “Nanostructured ZnO: from Monodisperse Nanoparticles to Nanorods”. *Journal of Crystal Growth* 305 (2007): 162-166.

10. M. Chase Jr, J. Curnutt, J. Downey Jr, R. McDonald, A. Syverud, E. Valenzuela, “JANAF Thermochemical Tables, 1982 Supplement”. *Journal of Physical and Chemical Reference Data* 11 (1982): 695-940.

11. C. Huang, Z. Deng, C. Yuan, M. Zhang, X. Wang, D. Yang, C. Tian, H. Wang, “Intensive Turbulence Eddy-Induced Piezoelectric Polarization Boosting Photocatalytic Hydrogen Production in ZnO/NF”. *Fuel* 406 (2026): 137058.

12. T. Kuru, A. Sarilmaz, E. Aslan, F. Ozel, I.H. Patir, “Rational Design of ZnO/SrTiO_3_ S-Scheme Heterojunction for Photo-Enhanced Piezocatalytic Hydrogen Production”. *Applied Surface Science* 682 (2025): 161704.

13. M. Lu, X. Wu, “Piezo-Photocatalytic Generation of H_2_ with MoS_2_/ZnO/CuFe_2_O_4_ Nanosheets”. *ACS Applied Nano Materials* 8 (2025): 5078-5091.

14. X. Dai, Z. Liu, Z. Zhao, J. Wang, H. Luo, L. Cheng, W. Chen, “Efficient Hydrogen Recovery from Wastewater Treatment by Piezo-Photocatalytic Heterostructure”. *Journal of Cleaner Production* 447 (2024): 141540.

15. Q. Jia, M. Li, W. Sun, “Defect Engineering for Enhancing the Piezoelectric Catalytic Activity of Bi_2_S_3_ for Hydrogen Production”. *Dalton Transactions* 54 (2025): 13703-13711.

16. P.-H. Wu, H.-Y. Lin, S.-N. Lai, Y.-C. Chen, J.M. Wu, “Electromechanically Responsive High-Entropy Ferroelectric Catalyst with Polar Nanoregions for Mechanically Activated Hydrogen Evolution”. *Nano Energy* 144 (2025): 111402.

17. W. Liu, S. Zhu, M. Ran, Z. Fu, M. Zhang, Y. Zhang, H. Wang, M. Xing, P. Fu, “Hydroenergy Inspiring Large‐Scale Piezoelectric Catalysis for Seawater Hydrogen Evolution”. *Angewandte Chemie International Edition* 64 (2025): e202504749.

18. N. Li, G. Zhao, Y. Wu, Y. Li, K. Zhao, W. Fu, S. Zhang, J. Ma, “Sn_3_O_4_ nanoflowers/Mn_0.5_Cd_0.5_S Heterojunction as Piezo-Photocatalyst for Hydrogen Evolution Reaction”. *ACS Applied Nano Materials* 7 (2024): 26952-26961.

19. K. Zhang, X. Sun, H. Hu, G. Yan, A. Qin, Y. Ma, H. Huang, T. Ma, “Defect Engineered Microcrystalline Cellulose for Enhanced Cocatalyst‐Free Piezo‐Catalytic H_2_ Production”. *Small* 19 (2023): 2304674.

20. S. Zhao, M. Liu, Y. Zhang, Z. Zhao, Q. Zhang, Z. Mu, Y. Long, Y. Jiang, Y. Liu, J. Zhang, “Harvesting Mechanical Energy for Hydrogen Generation by Piezoelectric Metal–Organic Frameworks”. *Materials Horizons* 9 (2022): 1978-1983.

21. X. Xue, B. Jiang, Z. Li, Y. Jiang, K. Zhang, Z. Ji, G. Song, C. Yang, “ZnO@PVDF-Nanopillar Membrane for Synergistic Mechano-Photocatalytic Dye Decomposition”. *Colloids and Surfaces A: Physicochemical and Engineering Aspects* 735 (2026): 139553.

22. F. Peng, X. Kai, Y. Bao, K. Tang, W. Wang, C. Wu, “Graphene Oxide Anchored ZnO Composites for Enhanced Piezoelectric Catalytic Nitrogen Fixation and Dye Degradation”. *Journal of Alloys and Compounds* 1016 (2025): 178942.

23. P.P. Gotipamul, S.A. Alqarni, S. Pandiaraj, M. Rathinam, S. Chidambaram, “Initiation of Piezoelectricity Expands the Photocatalytic H_2_ Production and Decomposition of Organic Dye through g-C_3_N_4_/Ag/ZnO Tri-Components”. *Materials Science for Energy Technologies* 7 (2024): 133-147.

24. P. Wang, W. Cai, F. Yu, P. Zhou, M. Lin, C. Lin, T. Lin, M. Gao, C. Zhao, X. Li, “Bi_0.5_Na_0.5_TiO_3_/ZnO Z-Scheme Heterojunction for Piezo-Photocatalytic Water Remediation: Mechanical Energy Harvesting and Energy Band Configuration”. *Chemosphere* 338 (2023): 139548.

25. P.P. Gotipamul, G. Vattikondala, K.D. Rajan, S. Khanna, M. Rathinam, S. Chidambaram, “Impact of Piezoelectric Effect on the Heterogeneous Visible Photocatalysis of g-C_3_N_4_/Ag/ZnO Tricomponent”. *Chemosphere* 287 (2022): 132298.

26. L. Meng, L. Zhou, C. Liu, H. Jia, Y. Lu, D. Ji, T. Liang, Y. Yuan, X. Zhang, Y. Zhu, “Synergistic Barium Titanate/MXene Composite as A High-Performance Piezo-Photocatalyst for Efficient Dye Degradation”. *Journal of Colloid and Interface Science* 674 (2024): 972-981.

27. X. Yan, S. Zhang, L. Pan, T. Ai, Z. Li, Y. Niu, “Synergetic Piezo-Photocatalytic Effect in NaNbO_3_/WO_3_ Photocatalyst for RhB Degradation”. *Inorganic Chemistry Communications* 158 (2023): 111510.

28. Q. Jing, Z. Liu, X. Cheng, C. Li, P. Ren, K. Guo, H. Yue, B. Xie, T. Li, Z. Wang, “Boosting Piezo-Photocatalytic Activity of BiVO_4_/BiFeO_3_ Heterojunctions through Built-in Polarization Field Tailoring Carrier Transfer Performances”. *Chemical Engineering Journal* 464 (2023): 142617.

29. X. Cheng, Z. Liu, Q. Jing, P. Mao, K. Guo, J. Lu, B. Xie, H. Fan, “Porous (K_0.5_Na_0.5_)_0.94_Li_0.06_NbO_3_-Polydimethylsiloxane Piezoelectric Composites Harvesting Mechanical Energy for Efficient Decomposition of Dye Wastewater”. *Journal of Colloid and Interface Science* 629 (2023): 11-21.

30. L. Li, W. Cao, J. Yao, W. Liu, F. Li, C. Wang, “Synergistic Piezo-Photocatalysis of BiOCl/NaNbO_3_ Heterojunction Piezoelectric Composite for High-Efficient Organic Pollutant Degradation”. *Nanomaterials* 12 (2022): 353.

31. H. Lei, H. Zhang, Y. Zou, X. Dong, Y. Jia, F. Wang, “Synergetic Photocatalysis/Piezocatalysis of Bismuth Oxybromide for Degradation of Organic Pollutants”. *Journal of Alloys and Compounds* 809 (2019): 151840.
